# Prevention of shivering post spinal anesthesia: Ondansetron vs. Nefopam ‒ a prospective randomized controlled trial

**DOI:** 10.1016/j.bjane.2025.844650

**Published:** 2025-06-11

**Authors:** Joanna Tohme, Joan Chehade, Hicham Abou Zeid, Rhea Mattar, Nicole Naccache, Khalil Jabbour, Mohammad Ali Ismail, Christine Dagher

**Affiliations:** Department of Anesthesia and Critical Care, Hôtel-Dieu de France Hospital, Saint-Joseph University, Beirut, Lebanon

**Keywords:** Nefopam, Ondansetron, Shivering, Spinal anesthesia

## Abstract

**Background:**

Post Spinal Anesthesia Shivering (PSAS) is common and linked to increased morbidity. While various methods exist to prevent it, no study has compared Nefopam and Ondansetron. This study aims to compare Ondansetron and Nefopam in preventing PSAS.

**Methods:**

A prospective, randomized, controlled, and double-blind trial was conducted in the operating room of a tertiary university hospital from April 5, 2021 to April 30, 2022. It included patients aged between 18 and 65 years scheduled for surgery under spinal anesthesia. Patients received either 8 mg of Ondansetron or 20 mg of Nefopam administered intravenously over 30 min before spinal anesthesia. Main outcome measures included the number and grades of shivering episodes post spinal anesthesia at 15-minute intervals until post-anesthesia care unit discharge. Secondary outcomes included number of episodes of hypotension, bradycardia, nausea and/or vomiting. Tympanic temperature and pain at the injection site were also recorded.

**Results:**

The study included 150 patients, evenly divided between the two groups. The Ondansetron group had a higher incidence of shivering compared to the Nefopam group (23.9 % vs. 16 %; *p* = 0.038), as well as higher incidences of hypotension (16 % vs. 5.3 %; *p* = 0.035) and bradycardia (13.3 % vs. 2.7 %; *p* = 0.016). The Ondansetron group had a significantly lower incidence of nausea and vomiting (12 % vs. 1.3 %; *p* = 0.010). More patients in the Nefopam group (45.3 %) reported pain during drug infusion.

**Conclusions:**

Nefopam seems to be more effective than Ondansetron in preventing PSAS with fewer cardiovascular side effects. However, Ondansetron reduces the incidence of nausea and vomiting and causes no pain during administration.

## Introduction

Spinal anesthesia is commonly used in many surgical procedures.^1^ This effective anesthesia technique is, however, associated with some undesirable side effects. Among them, shivering can affect up to 40 % to 60 % of patients.^2^^,^^3^ Shivers occur in response to disturbances of the homeostatic system triggered by spinal anesthesia. The underlying mechanism is lower limb vasodilation, inducing rapid heat loss and redistribution of body heat from the central to the peripheral compartment, thus resulting in hypothermia and shivering.^4^

Shivers are uncomfortable for patients and also challenging for anesthesiologists as they interfere with monitoring parameters. Moreover, they can lead to a cascade of physiological changes. Shivering increases metabolic activity, oxygen consumption, and induces arterial hypoxemia, potentially amplifying the risk of ischemic events, as well as, increasing intracranial and intraocular pressure, increasing cardiac output and peripheral vascular resistance, and inducing lactic acidosis.^5^, ^6^, ^7^ All these factors are associated with increased morbidity, especially in elderly and fragile patients.

Many non-pharmacological and pharmacological methods are available to prevent and treat Post Spinal Anesthesia Shivering (PSAS) such as Ondansetron, Pethidine or other opioids, Physostigmine, Nefopam, Ketamine, and Doxapram.^5^^,^^8^

Ondansetron, initially used to treat nausea and vomiting, has recently shown encouraging results in reducing PSAS by attenuating the drop in core temperature, a potential trigger for shivering.^9^^,^^10^ On the other hand, Nefopam, a non-opioid analgesic, has also demonstrated its effectiveness in preventing post spinal anesthesia shivers, with a distinct mechanism of action.^5^

To our knowledge, no prospective and randomized study has yet been conducted to specifically compare Nefopam with Ondansetron in PSAS prevention.

## Materials and methods

### Ethics

Ethical approval for this study (CEHDF 1589) was provided by the Ethical Committee of Hôtel-Dieu de France Hospital, Beirut, Lebanon (Chairperson Prof. Sami Richa) on September 24, 2020.

Written informed consent was obtained from all study participants. The Helsinki declarations of 1963 were considered: respect, confidentiality, and patient anonymity.

We conducted a prospective randomized, controlled, double-blind trial with 2 parallel groups comparing the impact of Ondansetron and Nefopam administration on the incidence and intensity of PSAS when used as prophylaxis in non-obstetric surgeries. This is a superiority trial between Nefopam and Ondansetron.

The study protocol (trial number: NCT04870541) was registered in ClinicalTrials.gov (https://clinicaltrials.gov./study/NCT04870541?term=NCT04870541&rank=1).

### Study estimates and sampling

We included patients aged between 18 and 65 years-old, who were scheduled for surgery under spinal anesthesia at Hôtel-Dieu de France, a university hospital in Beirut, Lebanon, between April 5, 2021, and April 30, 2022. The study was conducted in a small country, with minimal ethnic or geographic diversity.

Exclusion criteria were pregnancy, breastfeeding, presence of allergy to any of the drugs used, patients with long QT syndrome, renal or hepatic insufficiency, epilepsy or Parkinson's disease, glaucoma, or phenylketonuria.

### Randomization

After informed consent, patients were randomized into 2 groups:

• Group A: patients receiving 8 mg of Ondansetron diluted in 20 mL of 0.9 % Saline Solution administered intravenously over 30 min before spinal anesthesia.
• Group B: patients receiving 20 mg of Nefopam diluted in 20 mL of 0.9 % Saline Solution administered intravenously over 30 min before spinal anesthesia.

Whenever the patients reported pain with a score greater than 3 on a 10-point Numeric Rating Scale (NRS) during drug infusion, we decreased the speed of drug administration by half.

Randomization was performed using a computer-generated random number concealed in sealed opaque envelopes, which remained opaque even when held to the light. The random sequence was generated using *R* (package: randomizeR) and was prepared by an independent statistician who was not involved in participant recruitment or data collection. The patients were included in one of the two groups according to the randomization sequence. To minimize selection bias, the envelopes were sequentially numbered and opened only after participant enrollment, ensuring allocation concealment. Once the patient was recruited, the sealed envelope was opened by a single nurse who prepared the drugs and presented them as coded syringes. The nurse was not aware of the study protocol. The patient, the anesthesiologist in the Operating Room (OR), and in the Post Anesthesia Care Unit (PACU) were blinded to the content of the syringe.

### Procedure

After admission to the OR, routine standard monitoring was used in all patients in the form of non-invasive blood pressure, pulse oximetry and Electrocardiogram (ECG). Room temperature in the operating rooms was maintained at 20°‒22 °C.

The attending anesthesiologist in charge of patient anesthesia was blinded to the study drug and not involved in data acquisition. The study protocol drug was started immediately upon arrival to the operating room. Spinal anesthesia was done at either L2/L3, L3/L4 or L4/L5 interspace with 0.5 % hyperbaric or isobaric bupivacaine and Sufentanil 2.5 µg. After completion of spinal anesthesia, oxygen was administered via a nasal cannula (2 L/min) till the end of the procedure.

Intraoperatively, all patients were covered at the shoulder level with a forced air warming blanket started immediately after spinal anesthesia and until transfer to PACU. Tympanic temperature was monitored by Braun® thermoscan thermometer every 15 min, and hemodynamic parameters every 3 min, until motor blockade resolution.

### Data

Data entry was performed by an independent person and included demographic characteristics, types of surgery, characteristics of spinal anesthesia (drugs used and sensory blockade level) as well as:

• Number of episodes of shivering and their grades post spinal anesthesia until PACU discharge. Shivering was graded from 0 to 3: 0 = No shivering; 1 = visible tremors of head and neck with ECG modifications with no arm movement; 2 = visible tremors in more than one muscle group and 3 = intense shivering, tremors of the whole body.
• Number of episodes of hypotension (defined as Systolic Blood Pressure [SBP] < 90 mmHg or less than 25 % of baseline SBP) post spinal anesthesia until PACU discharge.
• Number of episodes of bradycardia (defined as Heart Rate [HR] < 50 min or less than 25 % of initial HR) post spinal anesthesia until PACU discharge.
• Number of episodes of nausea and/or vomiting intra and postoperatively.
• Monitoring of tympanic temperature every 15 min post spinal anesthesia and in PACU to detect hypothermia (defined as temperature lower than 35.5 degrees Celsius).
• Presence of pain at site of injection during intravenous study drug infusion. Pain is defined as a score greater than 3 on a 10-point NRS, where 0 represents no pain and 10 represents the worst possible pain.

### Outcomes

The primary outcome was comparing the incidence of PSAS, as well as the intensity of PSAS, in non-obstetric surgeries between patients receiving Ondansetron vs. Nefopam.

Secondary outcomes included evaluation of hemodynamic variations (hypotension and bradycardia), incidence of nausea and vomiting intraoperatively and in PACU, incidence of hypothermia intraoperatively and in PACU, and intensity of pain at site of injection during study drug infusion.

### Statistics

Distribution of continuous variables was checked using the Shapiro-Wilk normality test and visual inspection of Quantile-Quantile plots. Categorical data were presented as frequency, percentage, and 95 % Confidence Intervals. Continuous data that did not deviate from normality were presented as mean ± Standard Deviation (*m* ± SD); ordinal data and continuous data that significantly deviate from normality were presented as Median (Med) and interquartile range [Q1‒Q3]. Categorical data were compared using Fisher's exact test. Normally distributed data were compared using Student's *t*-test for independent samples. Non-normally distributed continuous data and ordinal data were compared using the Mann-Whitney non-parametric test. All tests are two-tailed, and the first type error risk is set at 5 % without adjustment for multiplicity.

## Results

**Figure 1:**
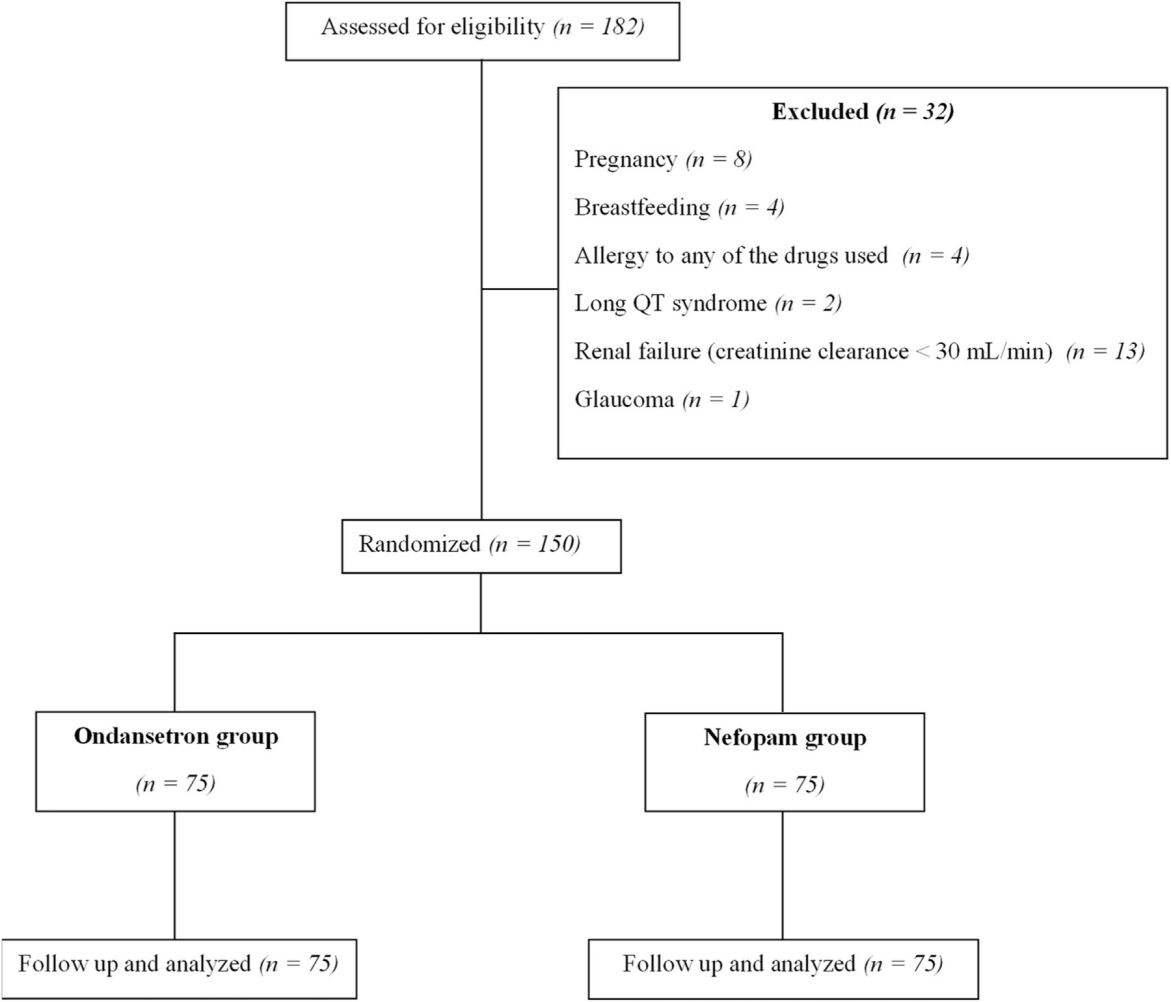
CONSORT flow diagram.

A consort flow diagram detailing the screening, recruitment, and analysis of the participants is shown in Figure 1. The study included 150 patients, evenly distributed between the 2 groups. All patients included were followed up in the OR and PACU (no exclusions after randomization). Demographic characteristics of patients are described in Table 1. No significant differences were observed regarding age, sex, and other relevant demographic parameters between the studied groups. Likewise, we noted a balanced distribution of surgeries between the 2 groups (Supplemental Table 1), as well as, homogeneity of the sensory blockade level, administered anesthetic drugs, and duration of spinal anesthesia (Table 2).

**Table 1:** Patients’ demographics across Nefopam and Ondansetron groups.

		Nefopam Group (*n* = 75)	Ondansetron Group (*n* = 75)
**Age (years, mean ± SD)**		45.1 ± 11.5	46.7 ± 13.1
**Weight (Kg, mean ± SD)**		76.1 ± 18.2	77.4 ± 17
**BMI (Kg/m², mean ± SD)**		26.5 ± 5.1	27.7 ± 5.6
**Sex female, n (%) [95% CI]**		49 (65.3 %) [54.1‒75.4 %]	48 (64 %) [52.8‒74.2 %]
**ASA, n (%) [95% CI]**	1	32 (42.7 %) [31.9‒54 %]	37 (49.3 %) [38.2‒60.5 %]
	2	41 (54.7 %) [43.4‒65.6 %]	37 (49.3 %) [38.2‒60.5 %]
	3	2 (2.7 %) [0.6‒8.3 %]	1 (1.3 %) [0.1‒6.1 %]
**APFEL Score, n (%) [95% CI]**	0	11 (14.7 %) [8.1‒23.9 %]	17 (22.7 %) [14.3‒33.1 %]
	1	37 (49.3 %) [38.2‒60.5 %]	24 (32 %) [22.3‒43.1 %]
	2	19 (25.3 %) [16.6‒36 %]	32 (42.7 %) [31.9‒54 %]
	3	6 (8 %) [3.4‒15.7 %]	2 (2.7 %) [0.6‒8.3 %]

CI, Confidence Interval; MWU, Mann-Whitney U; SD, Standard Deviation.

**Table 2:** Spinal anesthesia characteristics across Nefopam and Ondansetron groups.

		Nefopam Group (*n* = 75)	Ondansetron Group (*n* = 75)	p
**Sensory block level, n (%) [95% CI]**	T4	‒	1 (1.4 %) [0.1‒6.2 %]	0.941
	T5	2 (2.7 %) [0.6‒8.5 %]	‒	
	T6	4 (5.5 %) [1.9‒12.5 %]	2 (2.7 %) [0.6‒8.5 %]	
	T7	3 (4.1 %) [1.2‒10.6 %]	7 (9.6 %) [4.4‒17.9 %]	
	T8	5 (6.8 %) [2.7‒14.4 %]	8 (11 %) [5.3‒19.6 %]	
	T9	6 (8.2 %) [3.5‒16.2 %]	3 (4.1 %) [1.2‒10.6 %]	
	T10	51 (69.9 %) [58.7‒79.5 %]	49 (67.1 %) [55.8‒77.1 %]	
	T11	2 (2.7 %) [0.6‒8.5 %]	3 (4.1 %) [1.2‒10.6 %]	
**Duration of spinal anesthesia (min) [95% CI]**		90 [60‒120]	100 [60‒120]	0.720
**Hyperbaric Bupivacaine, n (%) [95% CI]**		62 (82.7 %) [72.9‒89.9 %]	66 (89.2 %) [80.6‒94.8 %]	0.347
**Bupivacaine dose (mg ± SD)**		8.6 ± 1	8.4 ± 1.1	0.397

CI, Confidence Interval; MWU, Mann-Whitney *U* test; min, minutes; SD, Standard Deviation.

### Post spinal anesthesia shivering

**Figure 2:**
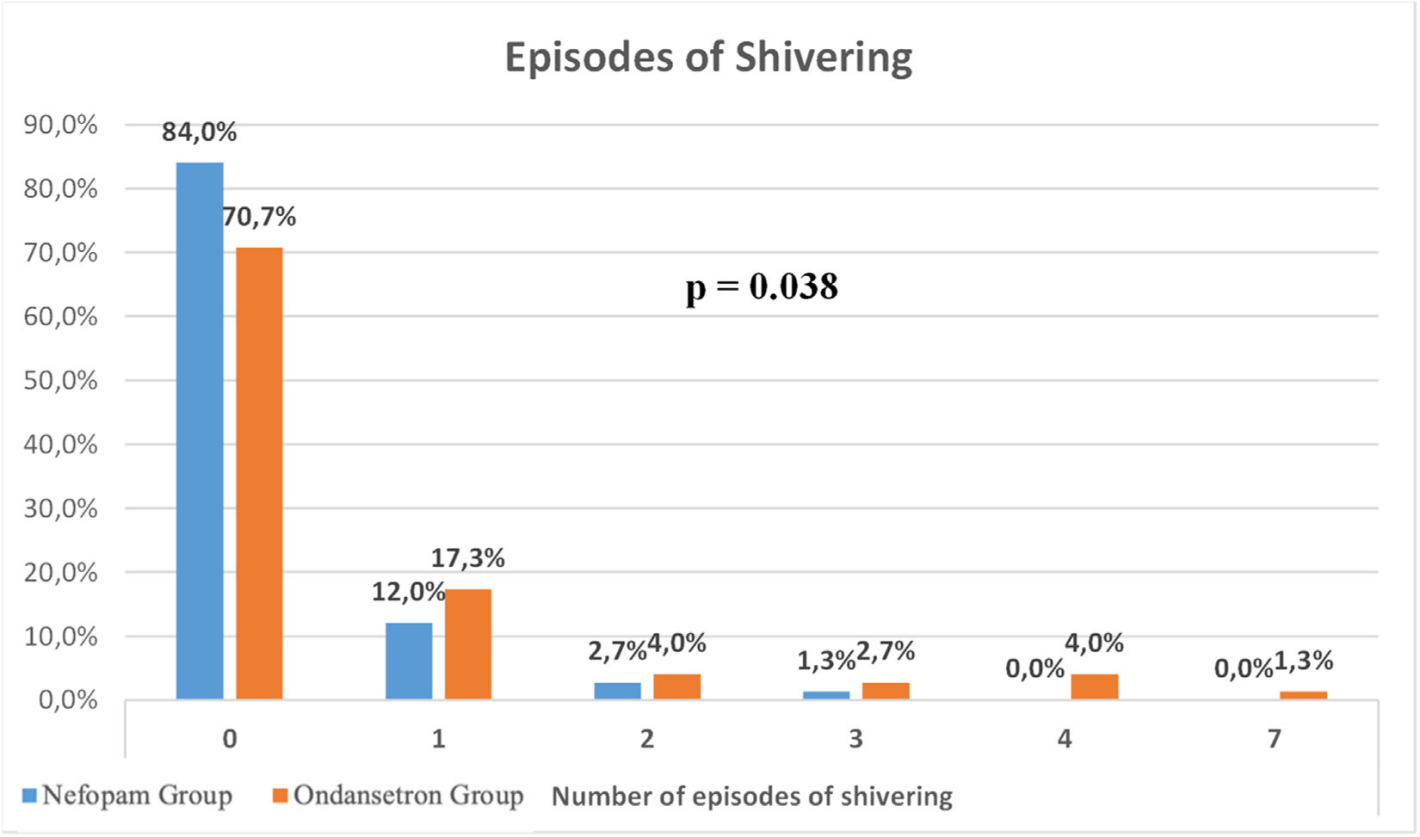
Number of shivering episodes across Nefopam and Ondansetron groups.

**Figure 3:**
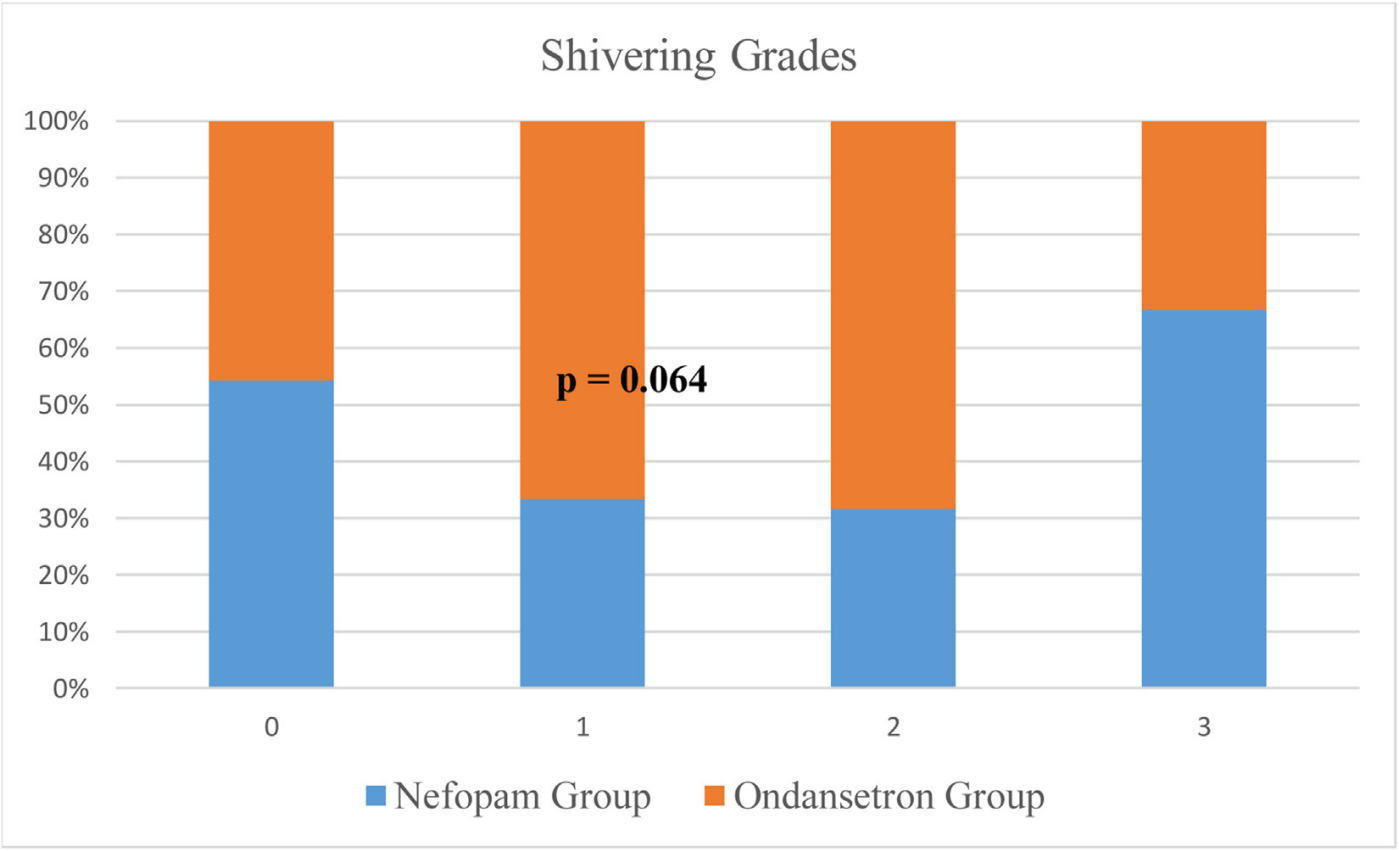
Grades of Shivering. Grades of shivering: 0 = No shivering; 1 = Visible tremors of head and neck with ECG modifications with no arm movement; 2 = Visible tremors in more than one muscle group and 3 = Intense shivering, tremors of the whole body.

Patients in the Ondansetron group showed a higher incidence of shivering compared with the Nefopam group (18 [23.9 %] vs. 12 [16 %] patients; *p* = 0.038) (Figure 2). The Risk Ratio (RR) with its corresponding 95 % Confidence Intervals (95 % CI) is 1.83 (95 % CI 0.98–3.43). This means that the incidence of shivering was 83 % higher in the Ondansetron group compared to the Nefopam group. Although higher grades of shivering were also observed in the Ondansetron group, this difference was not statistically significant (*p* = 0.064) (Figure 3).

### Cardiovascular effects and sensory levels

Significant differences were noted in cardiovascular responses between the two groups (Table 3). The Ondansetron group showed a higher incidence of hypotension episodes (12 [16 %] vs. 4 [5.3 %] patients; *p* = 0.035) and bradycardia episodes (10 [13.3 %] vs. 2 [2.7 %] patients; *p* = 0.016) compared with the Nefopam group, although sensory levels after spinal anesthesia were comparable between the two groups (*p* = 0.941).

**Table 3:** Drugs’ associated side effects.

		Nefopam Group (*n* = 75)	Ondansetron Group (*n* = 75)	p
**Hypotension episodes, n (%) [95% CI]**	**0**	71 (**94.7%**) [87.8‒98.2 %]	6 3 (**84%**) [74.5‒90.9 %]	**0.035**
	1	2 (2.7 %) [0.6‒8.3 %]	7 (9.3 %) [4.3‒17.5 %]	
	2	2 (2.7 %) [0.6‒8.3 %]	2 (2.7 %) [0.6‒8.3 %]	
	3	‒	1(1.3 %) [0.1‒6.1 %]	
	4	‒	2 (2.7 %) [0.6‒8.3 %]	
**Bradycardia episodes, n (%) [95% CI]**	**0**	73 (**97.3%**) [91.7‒99.4 %]	65 (**86.7%**) [77.6‒92.9 %]	**0.016**
	1	1 (1.3 %) [0.1‒6.1 %]	5 (6.7 %) [2.6‒14 %]	
	2	1 (1.3 %) [0.1‒6.1 %]	1 (1.3 %) [0.1‒6.1 %]	
	3	‒	2 (2.7 %) [0.6‒8.3 %]	
	4	‒	1 (1.3 %) [0.1‒6.1 %]	
	6	‒	1 (1.3 %) [0.1‒6.1 %]	
**Nausea and Vomiting episodes, n ( %) [95 % CI]**	**0**	66 (**88 %**) [79.2‒93.9 %]	74 (**98.7 %**) [93.9‒99.9 %]	**0.010**
	1	5 (6.7 %) [2.6‒14 %]	‒	
	2	4 (5.3 %) [1.8‒12.2 %]	1 (1.3 %) [0.1‒6.1 %]	
**Pain during drug infusion, n ( %) [95 % CI]**		34 (**45.3 %**) [34.4‒56.6 %]	4 (**5.3 %**) [1.8‒12.2 %]	**0.000**
**Hypothermia, n ( %) [95 % CI]**		32 (42.7 %) [31.9‒54 %]	31 (41.3 %) [30.7‒52.6 %]	1.000

CI, Confidence Interval; MWU, Mann-Whitney *U* test.

### Nausea, vomiting, and perioperative hypothermia

We noted a significant decrease in the incidence of nausea and vomiting in the Ondansetron group compared with the Nefopam group (9 [12 %] vs. 1 [1.3 %] patients; *p* = 0.010). The frequency of perioperative hypothermia was similar between the two groups (31 [41.3 %] in Ondansetron vs. 32 [42.7 %] patients in Nefopam group; *p* = 0.717) (Table 3).

### Reactions to the product and associated pain

A significantly higher percentage of patients in the Nefopam group (34 patients [45.3 %]) reported painful sensations during drug infusion compared with those in Ondansetron group (4 patients [5.3 %]) (*p* = 0.000).

## Discussion

Spinal anesthesia is a safe anesthetic technique practiced commonly worldwide.^1^ However, PSAS is a commonly encountered side effect. Multiple pharmaco‑therapeutic drugs have been studied for prevention of PSAS.^5^^,^^8^ Among them, Ondansetron and Nefopam have emerged as promising options. To our knowledge, this is the first study to compare the effect of these drugs on preventing PSAS.

The dose of 8 mg Ondansetron was selected based on prior studies demonstrating its efficacy in similar contexts. Kelsaka et al.^11^ showed that Ondansetron 8 mg intravenously administered immediately before spinal anesthesia had antishivering effects. The doses of Nefopam in the literature vary between 0.15 mg/kg and 0.2 mg/kg.^12^^,^^13^ We opted for a fixed dose of 20 mg, as it aligns with the standard practice in our institution for analgesia, ensuring consistency and practical applicability in our clinical setting.

In the present study, the incidence of PSAS was significantly reduced with Nefopam when compared with Ondansetron. Shivers’ intensity seemed also lower with Nefopam even though not statistically significant. The primary outcome included two independent variables: the incidence of PSAS and their intensity. Since these variables were analyzed separately and the results of shivering intensity showed no statistically significant difference, no correction was applied to the statistical tests. However, it is important to note that results associated with multiple analyses should be interpreted carefully.

Concerning side effects, Ondansetron was associated with more episodes of hypotension and bradycardia and Nefopam was associated with higher incidence of nausea, vomiting and pain during drug infusion. We did not compare the 2 groups of drugs to a placebo group, since it has been well established that both drugs are beneficial on preventing shivering after spinal anesthesia.^9^^,^^14^ Moreover, it is important to note that pethidine, the gold standard anti-shivering drug, as well as other opioid drugs could be associated with opioid related side effects as over-sedation, respiratory depression, nausea and vomiting, itching, constipation, and postoperative opioid induced hyperalgesia.^8^^,^^15^^,^^16^ The main advantage of both Ondansetron and Nefopam is that they are devoid of these adverse effects.

When it comes to anti-shivering effects, Nefopam has been described as causing a small increase in the core temperature by lowering the shivering threshold and without influencing sweating and vasoconstriction thresholds, therefore minimizing heat loss.^17^ Nefopam also affected thermoregulatory response via α2-adrenoceptors.^18^ Ondansetron has a central mechanism in reducing the shivering response by inhibition of serotonin reuptake at the level of the pre-optic anterior hypothalamic region. As a matter of fact, its anti-shivering effect is independent of the intraoperative core temperature, as observed by Powell and Buggy.^19^ Incidence of shivering across both groups in this study showed no correlation with the incidence of hypothermia, as hypothermia incidence was comparable between the 2 groups. This finding is comparable to other studies that found no correlation between shivering and hypothermia.^10^^,^^20^

The findings of the current trial go in agreement with results of other studies that concluded that prophylactic administration of Ondansetron showed a substantial reduction in the incidence and scores of shivering in both non-obstetric^14^^,^^21^ and obstetric surgeries.^22^ Likewise, many studies showed that the prophylactic administration of Nefopam decreased the incidence of PSAS.^5^^,^^16^^,^^23^

Our results highlight the difference in action mechanisms of both Ondansetron and Nefopam. In fact, Nefopam is a non-opioid, non-steroidal centrally acting analgesic. It acts by inhibiting the reuptake of serotonin, noradrenaline, and dopamine.^24^^,^^25^ It also possesses action on α2-adrenergic^26^ and is a noncompetitive NMDA receptor antagonist.^27^ Hence, it has sympathomimetic and anticholinergic effects, which explains the fewer episodes of hypotension and bradycardia described in our study. These results are concordant with other studies that described hemodynamic stability with the use of Nefopam.^16^^,^^23^ On the other hand, Ondansetron acts as a 5HT-3 (5-hydroxytryptamine-3) receptor antagonist, which explains its efficacy in reducing nausea and vomiting.^28^ This finding is comparable to other studies that found a decrease in nausea and vomiting with Ondansetron even at a lower dose of 4 mg.^9^^,^^10^ Even though, few studies described that Ondansetron may possess protective potentials against spinal anesthesia induced hypotension.^29^ Others concluded that Ondansetron had no actual capabilities to reduce the incidence of hypotension and shivering during cesarean section after spinal anesthesia, but could efficiently decrease incidence of nausea, vomiting, and bradycardia.^30^^,^^31^ In this study, results showed that Nefopam was superior to Ondansetron in reducing hypotension and bradycardia.

Finally, when it comes to pain during injection of the compared drugs, this study showed that 45 % of patients reported pain during infusion of Nefopam which was significantly higher than with Ondansetron (5.3 %). These results are in agreement with other studies that also described pain during infusion of Nefopam in patients under spinal anesthesia.^17^^,^^23^ It has been suggested that injection pain was associated with rapid increases in cerebral concentration of Nefopam.^32^

### Limitations

This study has a few limitations. First, it was conducted at a single center with a relatively small sample size, which may limit the generalizability of our findings. The sample size was calculated based on the formula: *n* = $\frac{\left(Z\alpha /2\;+\;Z\beta )2\;(p1\;(1\;-\;p1)\;+\;p2(1\;-\;p2)\right)}{(p1-p2)2}$, where p_1_ = 0.40 (baseline shivering rate, determined based on the review of literature) and p_2_ = 0.20 (we wanted to detect a 50 % decrease in the shivering rate). The sample size, adequate for primary outcomes, may be suboptimal for detailed secondary analyses. However, the authors believe that the prospective randomized double-blind design decreased the possibility of bias.

Second, the study population was limited to patients undergoing non-obstetric surgeries, so the findings may not be applicable to obstetric surgeries.

Third, a limitation of this study is the fixed dosing of Ondansetron (8 mg) and Nefopam (20 mg), which were selected based on commonly used clinical regimens and prior studies demonstrating their efficacy in similar contexts. However, different dosing strategies could potentially influence the outcomes, and we did not explore dose-response relationships, and this is an important consideration for future research.

Fourth, this study lacked quantification of administered fluids and the use of intravenous fluids. While our study aimed to reflect real-world clinical practice, we recognize that variations in fluid management may have affected hemodynamic outcomes. In future studies, we could consider standardized fluid administration protocols to better assess the independent effect of Nefopam and Ondansetron on hemodynamic stability. However, it is important to note that no difference in temperature was observated between the 2 groups.

## Conclusion

This study provides valuable insights into the comparative effectiveness of Ondansetron and Nefopam for PSAS prevention. While Nefopam demonstrates superior efficacy in preventing shivering with fewer cardiovascular side effects, Ondansetron offers advantages in reducing the incidence of nausea and vomiting with no pain during administration. Future research should explore larger, multicenter studies including obstetric surgeries to further elucidate whether different doses and rate of administration of both drugs impact PSAS and their side effects.

## Conflicts of interest

The authors certify that there is no conflict of interest with any financial organization regarding the material discussed in the manuscript.

## Appendix Supplementary materials

Image, application 1

## Footnotes

Supplementary material associated with this article can be found in the online version at doi:10.1016/j.bjane.2025.844650.
