# Supplementary material for: Prevention of shivering post spinal anesthesia: Ondansetron vs. Nefopam ‒ a prospective randomized controlled trial
Source: Braz J Anesthesiol. 2025 Jun 11;75(5):844650. doi: 10.1016/j.bjane.2025.844650 (PMC12268186; doi:10.1016/j.bjane.2025.844650)
Supplement: Supplementary file 1 [file mmc1.docx]

**BJAN-D-24-00690_Supplementary Material**

**Supplemental Table 1** Types of surgeries across Nefopam and Ondansetron groups.

|  | **Nefopam Group (n = 75) ( %)** | **Ondansetron Group (n = 75) ( %)** | **p** |
| --- | --- | --- | --- |
| **Gynecological Surgery** | 36 (48 %) | 34 (46.7 %) | 0.83 |
| **Orthopedic Surgery** | 36 (48 %) | 34 (46.7 %) |  |
| **Urologic Surgery** | 15 (20 %) | 20 (27.40 %) |  |
| **Visceral and Digestive Surgery** | 8 (10.60 %) | 8 (11 %) |  |
| **Vascular Surgery** | 0 (0 %) | 1 (1.40 %) |  |
